# UPSIT subitems may predict motor progression in Parkinson’s disease

**DOI:** 10.3389/fneur.2023.1265549

**Published:** 2023-10-23

**Authors:** Yu-Hsuan Lin, Ting-Chun Fang, Hsin-Bei Lei, Shih-Chi Chiu, Ming-Hong Chang, Yi-Jen Guo

**Affiliations:** ^1^The Department of Neurological Institute, Taichung Veterans General Hospital, Taichung, Taiwan; ^2^Department of Post-Baccalaureate Medicine, College of Medicine, National Chung Hsing University, Taichung, Taiwan; ^3^Brain and Neuroscience Research Center, College of Medicine, National Chung Hsing University, Taichung, Taiwan

**Keywords:** Parkinson’s disease, hyposmia, UPSIT, odor emotional valence, motor progression

## Abstract

**Background:**

The relationship between hyposmia and motor progression is controversial in Parkinson’s disease (PD). The aim of this study was to investigate whether preserved identification of Chinese-validated University of Pennsylvania Smell Identification Test (UPSIT) odors could predict PD motor progression.

**Methods:**

PD patients with two consecutive clinical visits while taking medication were recruited. Based on mean changes in Movement Disorder Society Unified Parkinson’s Disease Rating Scale part 3 score and levodopa equivalent daily dosage, the participants were categorized into rapid progression, medium progression, and slow progression groups. Odors associated with the risk of PD motor progression were identified by calculating the odds ratios of UPSIT item identification between the rapid and slow progression groups. Receiver operating characteristic curve analysis of these odors was conducted to determine an optimal threshold for rapid motor progression.

**Results:**

A total of 117 PD patients were screened for group classification. Preserved identification of neutral/pleasant odors including banana, peach, magnolia, and baby powder was significantly correlated with rapid motor progression. The risk of rapid progression increased with more detected risk odors. Detection of ≥1.5 risk odors could differentiate rapid progression from slow progression with a sensitivity of 85.7%, specificity of 45.8%, and area under the receiver operating characteristic curve of 0.687.

**Conclusion:**

Preserved identification of neutral/pleasant odors may help to predict PD motor progression, and detection of ≥1.5 UPSIT motor progression risk odors could improve the predictive power. In PD patients with a similar level of motor disability during initial screening, preserved pleasant/neutral odor identification may imply relatively better cortical odor discriminative function, which may suggest the body-first (caudo-rostral) subtype with faster disease progression.

## 1. Introduction

Olfactory dysfunction is an important early non-motor symptom of Parkinson’s disease (PD) (1). Hyposmia typically precedes the presence of the cardinal motor features of PD by several years, and affects up to 96% of patients (2). The literature supports that impaired olfaction is caused by the spread of Lewy body pathology to the olfactory bulb and anterior olfactory nucleus (3), as well as the degeneration of cholinergic circuits (4, 5). While hyposmia has been suggested to be a biomarker for cognitive impairment (6), its association with parkinsonian motor deficits remains controversial (7–9). Malasa et al. proposed that both the odor identification and the odor discrimination, as assessed through the Sniffin’s Sticks test, were associated with motor deficits (7). Conversely, in the review article written by Ercoli T, et al., five studies could not demonstrate correlation between olfactory dysfunction and severity of motor symptoms (10). The disparities in the outcomes of these studies may stem from the utilization of different olfactory assessments, suggesting the potential for identification of more specific odors associated with clinical symptoms of PD. The University of Pennsylvania Smell Identification Test (UPSIT) is the most widely used tool to evaluate PD olfactory deficits (11). A few abbreviated UPSIT subsets have been proposed to have potential in identifying hyposmia in PD (12, 13). Currently, there is no existing literature exploring the correlation between the odor discrimination of different items in the UPSIT and the motor progression in individuals with PD. In recent years, researchers propose that the propagation of Lewy pathology in PD occurs through two distinct patterns: the body-first model and the brain-first model. The body-first subtype PD patients, when compared to the brain-first subtype ones, tend to demonstrate a more rapid rate of motor symptom progression (14). As higher order olfactory processing, including odor identification and valence judgment, is influenced by brain regions including the amygdala, hippocampus, thalamus, and orbitofrontal cortex (15), it is plausible that the body-first and brain-first subtypes may yield divergent outcomes within the context of UPSIT, owing to the involvement of varying brain regions during the disease course (14). The aim of this study was to determine the discriminant odorants of UPSIT, especially those related to the emotional valences, in predicting PD motor progression.

## 2. Materials and methods

### 2.1. Subjects

In this retrospective study, PD patients were recruited from the Center for Parkinson and Movement Disorders of Taichung Veterans General Hospital between October 2016 and April 2022. All patients met the clinically probable PD diagnostic criteria of the International Parkinson and Movement Disorder Society Clinical Diagnostic Criteria for Parkinson’s Disease (16). Clinical evaluations were conducted while the patients were taking medication and included the following: UPSIT (17), Movement Disorder Society Unified Parkinson’s Disease Rating Scale (MDS-UPDRS) (18), Hoehn and Yahr (HY) Scale (19), Montreal Cognitive Assessment (MoCA) (20), and Beck’s Depression Inventory-II (BDI-II) (21). Levodopa equivalent daily dosage (LEDD) (22) was calculated for all participants. In the final analysis, only those who completed a second MDS-UPDRS evaluation with a minimum interval of 6 months from the first evaluation were enrolled. This study was approved by the Institutional Review Board of Taichung Veterans General Hospital (CE22189B-1). To safeguard the patients’ privacy, all personal information was encrypted.

### 2.2. Olfactory testing

A Chinese version of the UPSIT was used to evaluate olfactory identification function. The test consists of the following 40 items: pizza, bubble gum, methanol, cherry, motor oil, mint, banana, sandalwood, leather, coconut, onion, fruit juice, licorice, fish, coffee, gasoline, strawberry, cedar, chocolate, rubber tire, lilac, turpentine, peach, root beer, jasmine, pineapple, grapefruit, orange, magnolia, watermelon, paint thinner, baby powder, smoke, pine, grape, lemon, soap, natural gas, rose, and peanut (17). A sniff strip on each page was scratched which released the embedded odorant. The participants were then asked to identify the correct odor from four choices (23).

### 2.3. Study design

**Figure 1:**
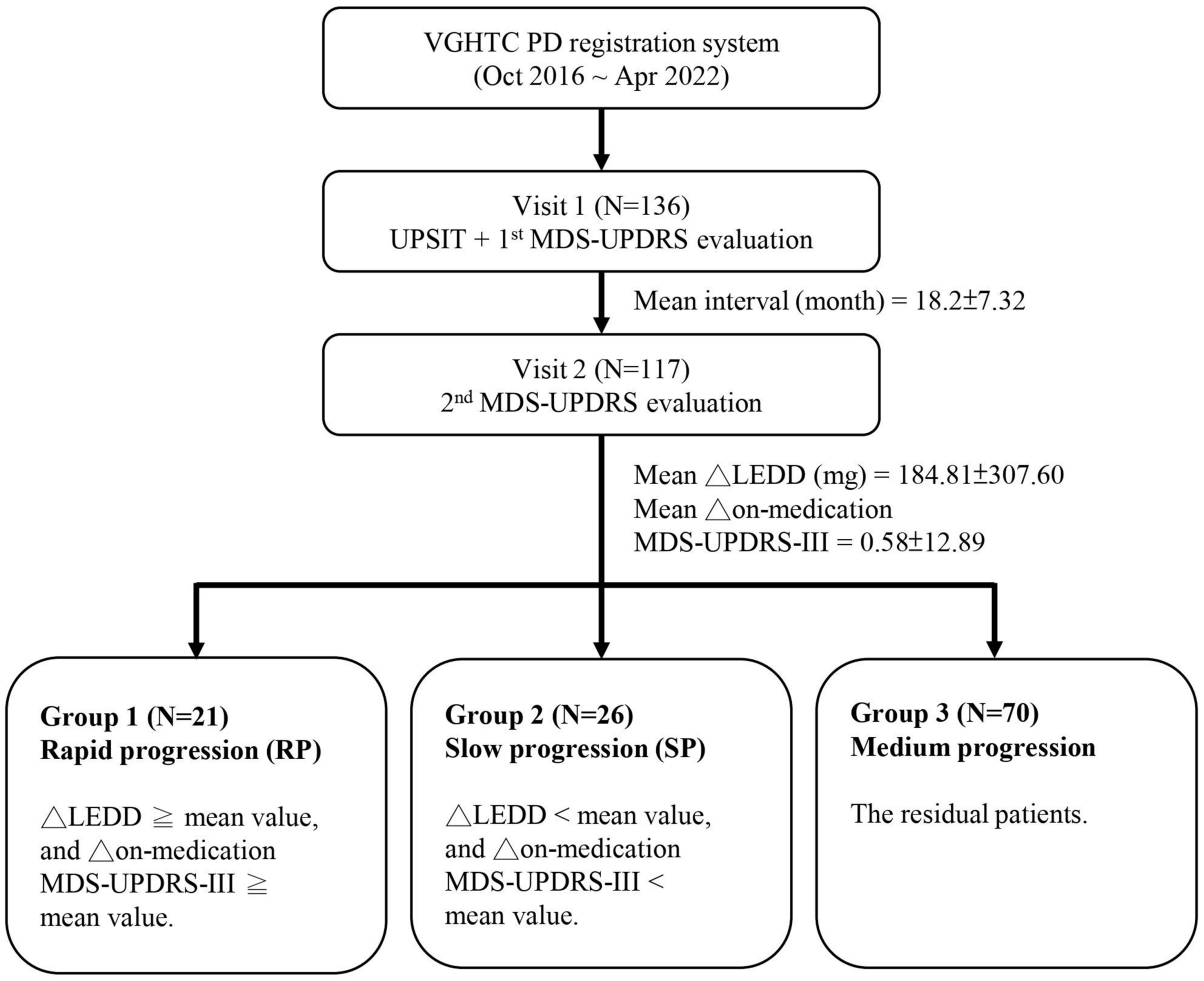
Schematic of the study design. VGHTC, Taichung Veterans General Hospital; PD, Parkinson’s disease; UPSIT, University of Pennsylvania Smell Identification Test; MDS-UPDRS, Movement Disorder Society Unified Parkinson’s Disease Rating Scale; MDS-UPDRS-III, MDS-UPDRS part 3 score; ∆, changes between two evaluations; LEDD, levodopa equivalent daily dose.

Based on the mean changes in MDS-UPDRS part 3 (MDS-UPDRS-III) score and LEDD between the two clinical evaluations, the enrolled PD patients were categorized into three groups for analysis. The rapid progression group was defined as those with changes in both LEDD and MDS-UPDRS-III score higher than the mean. Conversely, the slow progression group was defined as those with changes in both LEDD and MDS-UPDRS-III score lower than the mean. The remaining subjects were classified into the medium progression group (Figure 1).

### 2.4. Statistical analysis

All data were analyzed using SPSS version 23.0 (IBM Inc., Armonk, NY). For demographic data analysis, the Kruskal-Wallis test was used for continuous variables, and the chi-square test was used for categorical variables. In investigating the disparities between the rapid progression group and slow progression group regarding the accurate identification of individual item in the UPSIT, the chi-square test was applied. The odds ratio (OR) between the two groups was calculated, with the significance of the result determined through either the Pearson chi-square test or the Fisher’s exact test. Furthermore, multivariate logistic regression analysis was employed to investigate the association between the risk odors and motor progression, while accounting for potential confounding factors including age, sex, disease duration of PD, and LEDD. A value of *p*<0.05 was considered statistically significant. UPSIT items with an OR > 1 and a statistically significant value of p were considered as UPSIT motor progression risk odors. Following the identification of risk odors, a chi-square test was utilized to examine the differences in the accurate identification of varying numbers of risk odors between the rapid progression and slow progression groups. For instance, the comparison included the accurate identification of one risk odor versus an inability to identify any risk odors, as well as the accurate identification of two risk odors versus an inability to identify any risk odors, and so forth. The ORs were calculated. Finally, numbers of risk odors identified by the PD patients during initial assessment and their outcomes as rapid or slow progression group during the follow-up period were combined to construct a receiver operating characteristic (ROC) curve. The Youden index was applied to determine the ideal number of detected UPSIT risk odors, serving as the threshold for predicting PD motor progression. Furthermore, the area under the ROC curve was calculated to quantify the discriminatory capacity of the UPSIT motor progression risk odors.

## 3. Results

### 3.1. Baseline demographics

A total of 117 PD patients were enrolled for the final analysis. The demographic characteristics of our PD cohort were as follows: mean age 65.0 ± 8.9 years, mean disease duration 4.1 ± 4.0 years, average interval between the two clinical visits 18.2 months, mean MDS-UPDRS score 49.6 ± 20.5, mean MDS-UPDRS-III score 30.3 ± 12.8, and mean LEDD 537.7 ± 414.9 mg. In terms of interval changes, there was a mean change in MDS-UPDRS-III score of 0.58 ± 12.98, and a mean change in LEDD of 184.81 ± 307.6 mg (Figure 1).

The demographic data of the rapid, medium and slow progression groups are summarized in Table 1. With the exception of MDS-UPDRS part 2 (MDS-UPDRS-II) score (mean score of 8.81 ± 7.03 in the rapid progression group, 9.94 ± 6.57 in the medium progression group, and 6.35 ± 5.56 in the slow progression group, *p* = 0.018) and MDS-UPDRS part 4 (MDS-UPDRS-IV) score (mean score of 2.29 ± 3.55 in the rapid progression group, 1.03 ± 2.07 in the medium progression group, and 0.46 ± 1.84 in the slow progression group, *p* = 0.031), there were no statistically significant differences among the groups in terms of other baseline demographic characteristics, including age, gender, education, age at onset, disease duration, follow-up interval, LEDD, HY stage, MoCA, BDI-II, UPSIT, MDS-UPDRS, MDS-UPDRS part 1, and MDS-UPDRS-III scores.

**Table 1:** The demographic characteristics of the patients with Parkinson’s disease.

	Rapid progression (*N* = 21)	Medium progression (*N* = 70)	Slow progression (*N* = 26)	*p*-value
Age, years (Mean ± SD)	63.71 ± 10.52	64.56 ± 7.91	67.12 ± 9.89	0.519
Gender, Male, (N, (%))	13 (61.9%)	47 (67.1%)	14 (53.8%)	0.232
Education, years (Mean ± SD)	10.52 ± 5.01	10.34 ± 4.50	10.08 ± 4.39	0.959
Age at onset, years (Mean ± SD)	60.14 ± 8.13	62.69 ± 2.45	63.00 ± 0.00	0.46
Disease duration, years (Mean ± SD)	4.71 ± 4.00	4.05 ± 3.07	3.81 ± 6.03	0.201
Follow-up interval, months (Mean ± SD)	21.05 ± 8.17	18.07 ± 7.43	16.00 ± 5.58	0.063
MoCA (Mean ± SD)	25.24 ± 4.78	25.59 ± 3.81	25.12 ± 4.03	0.831
BDI-II (Mean ± SD)	11.24 ± 5.06	11.40 ± 9.06	8.31 ± 7.52	0.144
UPSIT (Mean ± SD)	17.48 ± 6.36	17.74 ± 5.97	14.88 ± 6.59	0.205
LEDD, mg (Mean ± SD)	574.11 ± 361.25	551.34 ± 432.46	471.77 ± 414.89	0.409
MDS-UPDRS total (Mean ± SD)	45.86 ± 18.22	51.36 ± 21.63	47.92 ± 19.20	0.515
MDS-UPDRS part1 (Mean ± SD)	8.81 ± 5.12	9.83 ± 5.69	8.04 ± 5.32	0.371
MDS-UPDRS part2 (Mean ± SD)^a^	8.81 ± 7.03	9.94 ± 6.57	6.35 ± 5.56	0.018^a^
MDS-UPDRS part3 (Mean ± SD)	25.95 ± 11.24	30.56 ± 13.29	33.08 ± 12.25	0.167
MDS-UPDRS part4 (Mean ± SD)^b^	2.29 ± 3.55	1.03 ± 2.07	0.46 ± 1.84	0.031^b^
Hoehn and Yahr Scale (Mean ± SD)	2.10 ± 0.63	2.16 ± 0.67	2.04 ± 0.34	0.614

SD, standard deviation; MoCA, Montreal Cognitive Assessment; BDI-II, Beck’s Depression Inventory-II; UPSIT, University of Pennsylvania Smell Identification Test; LEDD, levodopa equivalent daily dose; MDS-UPDRS, Movement Disorder Society Unified Parkinson’s Disease Rating Scale.

a : medium progression vs. slow progression, *p* = 0.014.

b : rapid progression vs slow progression, *p* = 0.035.

### 3.2. Identification of UPSIT motor progression risk odors

All of the 40 UPSIT items were analyzed to determine which odors could predict motor progression in the PD patients. Preservation of four specific odors had significantly higher ORs when comparing the rapid progression group to the slow progression group: banana (OR = 3.619, 95% CI = 1.064–12.306, *p* = 0.036), peach (OR = 4.62, 95% CI = 1.262–16.917, *p* = 0.017), magnolia (OR = 4.411, 95% CI = 1.282–15.174, *p* = 0.016), and baby powder (OR = 3.733, 95% CI = 1.053–13.242, *p* = 0.037) (Table 2). These four odors were classified as being motor progression risk odors in our PD cohort. The result remained statistically significant in multivariate logistic regression adjusting for potential confounding factors, including age, sex, disease duration, and LEDD (OR = 3.619, *p* = 0.039 for banana; OR = 4.620, *p* = 0.021 for peach, OR = 4.411, *p* = 0.019 for magnolia; OR = 3.733, *p* = 0.041 for baby powder).

**Table 2:** Odds ratios of preserved UPSIT item identification in predicting motor symptom progression in PD (rapid progression versus slow progression).

UPSIT item	OR	95%CI	*p*-value	UPSIT item	OR	95%CI	*p*-value
1. Pizza	0.351	(0.063, 1.958)	0.269	21. Lilac	3.643	(0.959, 13.836)	0.051
2. Bubble gum	0.615	(0.191, 1.981)	0.414	22. Turpentine	1.455	(0.454, 4.664)	0.528
3. Menthol	1.467	(0.444, 4.846)	0.529	23. *Peach*	4.62	(1.262, 16.917)	0.017
4. Cherry	0.579	(0.095, 3.520)	0.678	24. Root beer	1.393	(0.432, 4.490)	0.579
5. Motor oil	0.988	(0.229, 4.264)	1	25. Jasmine	0.839	(0.259, 2.718)	0.77
6. Mint	1.896	(0.588, 6.112)	0.282	26. Pineapple	2.5	(0.711, 8.784)	0.148
7. *Banana*	3.619	(1.064, 12.306)	0.036	27. Grapefruit	1.333	(0.358, 4.965)	0.668
8. Sandalwood	2	(0.557, 7.177)	0.284	28. Orange	0.944	(0.28, 3.183)	0.927
9. Leather	1.283	(0.405, 4.062)	0.671	29. *Magnolia*	4.411	(1.282, 15.174)	0.016
10. Coconut	0.848	(0.225, 3.196)	0.808	30. Watermelon	0.682	(0.206, 2.253)	0.529
11. Onion	1.563	(0.455, 5.362)	0.477	31. Paint thinner	0.807	(0.254, 2.566)	0.716
12. Fruit juice	2.051	(0.577, 7.290)	0.263	32. *Baby powder*	3.733	(1.053, 13.242)	0.037
13. Licorice	1.1	(0.348, 3.477)	0.871	33. Smoke	3	(0.903, 9.963)	0.069
14. Fish	0.383	(0.037, 3.984)	0.617	34. Pine	2.986	(0.883, 10.096)	0.074
15. Coffee	1.67	(0.486, 5.747)	0.414	35. Grape	1.719	(0.398, 7.431)	0.486
16. Gasoline	0.167	(0.018, 1.513)	0.112	36. Lemon	1.042	(0.268, 4.045)	1
17. Strawberry	0.917	(0.181, 4.638)	1	37. Soap	1.563	(0.455, 5.362)	0.477
18. Cedar	0.59	(0.163, 2.142)	0.421	38. Natural gas	1.5	(0.472, 4.771)	0.491
19. Chocolate	1.467	(0.444, 4.846)	0.529	39. Rose	0.286	(0.052, 1.557)	0.16
20. Rubber tire	0.807	(0.254, 2.566)	0.716	40. Peanut	1.059	(0.314, 3.568)	0.927

UPSIT, University of Pennsylvania Smell Identification Test; OR, odds ratio; CI, confidence interval.

### 3.3. Threshold of the number of UPSIT motor progression risk odors to predict PD motor progression

**Figure 2:**
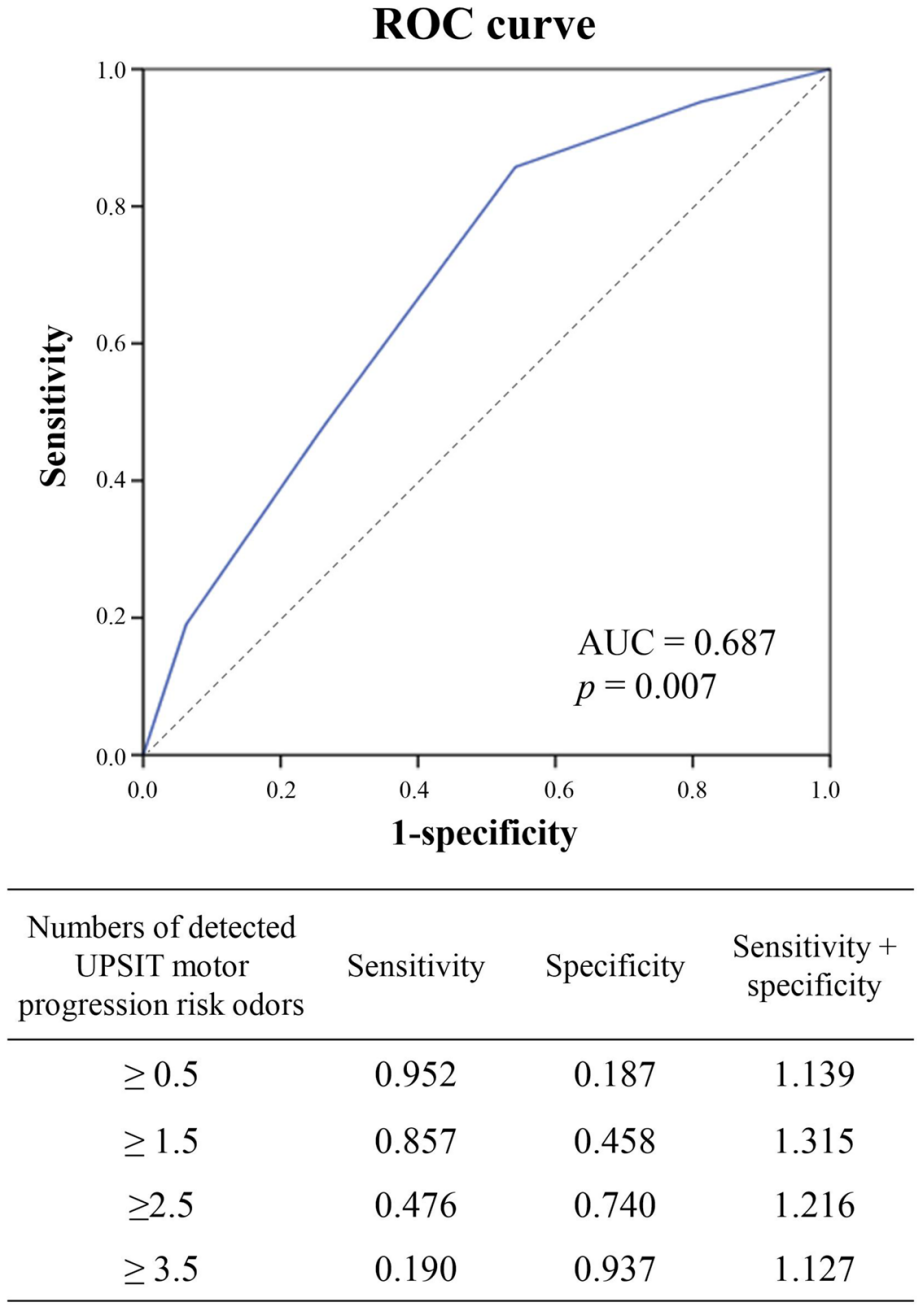
Receiver operating characteristic (ROC) curve analysis to determine the optimal cutoff value for the number of UPSIT risk odors to predict motor progression in the patients with PD (area under the ROC curve = 0.687, *p* = 0.007). Preservation of more than 1.5 risk odors had better sensitivity + specificity to predict motor progression, which is shown in the ROC curve figure. UPSIT, University of Pennsylvania Smell Identification Test; UPSIT risk odors: banana, peach, magnolia, baby powder.

To optimize the predictive power for PD motor progression of the UPSIT motor progression risk odors (baby powder: sensitivity 76.2%, specificity 53.8%, positive predictive value (PPV) 57.1% and negative predictive value (NPV) 73.7%; peach: sensitivity 52.4%, specificity 77.8%, PPV 68.6% and NPV 67.7%; banana: sensitivity 57.1%, specificity 73.1%, PPV 63.2% and NPV 67.9%; magnolia: sensitivity 61.9%, specificity 73.1%, PPV 65% and NPV 70.1%), we combined these four progression risk odors in further analysis to determine an ideal prediction threshold (Table 3 and Figure 2).

**Table 3:** Odds ratios of the number of detected UPSIT motor progression risk odors in predicting PD motor progression (rapid progression versus progression).

Detected number of UPSIT motor progression risk odors	*N*	OR	*p*-value	95% CI
1 vs. 0	19	2.571	0.582	(0.192, 34.473)
2 vs. 0	25	10.286	0.04	(1.030, 102.753)
3 vs.0	18	27	0.013	(1.979, 368.383)
4 vs. 0	15	36	0.017	(1.772, 731.562)
≥1 vs. 0	47	1.727	0.678	(0.284, 10.501)
≥2 vs. 0	38	16.2	0.003	(1.785, 147.065)
≥3 vs. 0	23	30	0.003	(2.626, 342.734)

UPSIT, University of Pennsylvania Smell Identification Test; N, number of patients; OR, odds ratio; CI, confidence interval.

The risk of motor progression notably increased as the number of detected UPSIT motor progression risk odors increased compared to being unable to detect any of these odors (two versus no risk odor detection: OR: 10.286, 95% CI = 1.030 to 102.753, *p* = 0.04; three versus no risk odor detection: OR: 27, 95% CI = 1.979 to 368.383, *p* = 0.013; four versus no risk odor detection: OR: 36, 95% CI = 1.772 to 731.562, *p* = 0.017; two or more versus no risk odor detection: OR: 16.2, 95% CI = 1.785 to 147.065, *p* = 0.003; three or more versus no risk odor detection: OR: 30, 95% CI = 2.626 to 342.734, *p* = 0.003) (Table 3).

To determine the optimal threshold of the number of UPSIT motor progression risk odors to predict PD motor progression, ROC curve analysis was conducted (Figure 2). The results indicated that detection of ≥1.5 UPSIT motor progression risk odors was the optimal cutoff value to predict PD motor progression considering both the sensitivity and specificity (sensitivity: 85.7%, specificity: 45.8%, area under the ROC curve: 0.687).

## 4. Discussion

In the current study, preserved identification of banana, peach, magnolia, and baby powder odors was significantly correlated with rapid motor progression in our PD cohort, and therefore these four odors were defined as UPSIT motor progression risk odors. In addition, the patients who were able to recognize more risk odors had a greater risk of rapid disease progression. Further ROC curve analysis suggested the cut-off value for predicting rapid motor progression with more than 1.5 risk odors detection having a sensitivity of 0.875, and for more than 2.5 risk odors identification showing a specificity of 0.74.

Previous studies investigating the relationship of motor symptoms with hyposmia in patients with PD have reported inconsistent results (7, 8, 24). Some cross-sectional studies have reported associations between olfactory deficits, including impaired odor discrimination or identification, with motor symptoms (7, 25–28), and negative correlations between the severity of hyposmia with the binding of striatal dopamine transporter (25, 27). In addition, lower odor discrimination scores have been associated with an increased duration of disease (29), and hyposmic PD patients have been reported to have faster disease progression (28, 30). Most researchers have focused on developing a shorter version of the UPSIT to aid in the diagnosis of hyposmia in PD (12, 13, 31, 32). In this study, we aimed to evaluate which, if any, items of the Chinese-validated UPSIT could predict the rate of motor progression. Among the 40 odors, correctly identifying banana, peach, magnolia, and baby powder odors was significantly associated with rapid motor progression.

In this study, the patients with preserved identification of neutral and pleasant odors showed a faster progression of motor symptoms. Odorant sensory input enters the primary olfactory cortex through the olfactory bulb (33). Subsequent olfactory processing, such as odor identification and valence judgment, is modulated by higher odor brain regions involving the amygdala, hippocampus, thalamus, and orbitofrontal cortex (15). Previous studies have indicated that the brain structures responsible for processing pleasant odor identification may overlap with those involved in motor control (34). A functional magnetic resonance imaging study demonstrated that pleasant odor stimulation activated the striatum and left inferior frontal gyrus in PD patients compared to healthy controls, whereas hypoactivation in the ventral striatum was seen in PD patients exposed to unpleasant odors (15). The inferior frontal gyrus has been shown to be involved in inhibitory control and motor learning during fine finger movements in PD patients (34). The recently proposed PD subtypes of caudo-rostral progression (body-first) and amygdala-centered (brain-first) Lewy body pathology exhibit different disease progression rates (14). Our findings suggest that PD patients who retain the ability to identify pleasant odors may be more likely to follow a caudo-rostral spread of Lewy body pathology due to the relatively preserved brain regions modulating olfactory processing. Consequently, these patients may tend to have a faster progression of motor symptoms.

There are several limitations to this study. First, there is no established standardization for categorizing rapid and slow progression in PD during on-medication status. Limited previous research from Tsiouris et al. used the concept of the top n-percentage range of the distribution to define rapid versus slow disease progression during serial clinical evaluations while PD patients in off-medication status (30). To address this challenge, we defined patients with a greater increase in LEDD and a more pronounced decline in MDS-UPDRS-III scores as ‘rapid progression,’ while those with a lesser increase in LEDD and a milder decline in MDS-UPDRS-III scores were categorized as “slow progression.” This approach was designed to ensure that the ‘rapid progression’ group represents patients who experience a comparatively faster deterioration in motor deficits. This led to difficulties in classifying many patients (*N* = 70) into the rapid or slow progression groups, resulting in fewer cases available for the final analysis. Since this is a retrospective study from clinical registration platform, it would be difficult to ask all patients stopping medication for evaluation considering the safety issues and patients’ willingness, and the primary aim of our study is to uncover potential indicators of disease progression from the initial UPSIT assessment during regular clinical practice as a pilot study in nature. Previous studies indicate that medication for PD does not significantly alter the outcomes of olfactory assessment tests (35, 36). The multivariate logistic regression analysis considering LEDD as the cofounding factor also did not affect the statistical significance of predicting motor progression using the identified UPSIT risk odors in our study. As a databank accumulated from a single medical center within a relative short period of time, we acknowledge the limited data number for further analysis, including patients with repeated UPSIT and MDS-UPDRS evaluations, at the current moment. Second, the demographic data indicated that the PD patients in the rapid progression group had higher MDS-UPDRS-II and MDS-UPDRS-IV scores. Nevertheless, there were no statistically significant differences in HY stage, MDS-UPDRS score, MDS-UPDRS-III score, LEDD, and disease duration among the three groups. This suggests that the baseline motor severity was consistent across the study groups. Third, although we thoroughly reviewed the medical records of all participants and excluded those with active rhinopathies, not every PD patient in our cohort underwent comprehensive rhinal examinations, which could have potentially affected the results of the olfactory tests. However, the number of patients with rhinopathies did not differ significantly among the three groups. In addition, since odor identification is controlled by the secondary olfactory cortex, we believe that the relationship between selective odor identification and PD motor progression remains meaningful. Since this study is pilot in nature with recruitment from single center and limited patient numbers, it is worthwhile to validate our findings in further investigations encompassing a multi-center, extensive scale, and inclusion of healthy controls.

## 5. Conclusion

Preservation of neutral/pleasant odor identification (banana, peach, magnolia, and baby powder odors in the UPSIT) may serve as a predictor of PD motor progression. Furthermore, detection of ≥1.5 UPSIT motor progression risk odors improved the predictive power. In PD patients with a similar level of motor disability, those with preserved pleasant/neutral odor identification may imply relatively better cortical odor discriminative function, which may in turn suggest that these patients have the body-first (caudo-rostral) subtype with rapid disease progression.

## Data availability statement

The raw data supporting the conclusions of this article will be made available by the authors, without undue reservation.

## Ethics statement

The studies involving humans were approved by the Institutional Review Board of Taichung Veterans General Hospital (CE22189B-1). The studies were conducted in accordance with the local legislation and institutional requirements. Written informed consent for participation was not required from the participants or the participants' legal guardians/next of kin in accordance with the national legislation and institutional requirements.

## Author contributions

Y-HL: Conceptualization, Formal analysis, Methodology, Writing – original draft. T-CF: Conceptualization, Methodology, Writing – review & editing. H-BL: Data curation, Formal analysis, Writing – review & editing. S-CC: Data curation, Formal analysis, Writing – review & editing. M-HC: Data curation, Supervision, Writing – review & editing. Y-JG: Conceptualization, Supervision, Writing – review & editing.
